# Two Cases of Angular Curvature, Supervening upon Lateral Curvature of the Spine

**Published:** 1866-08

**Authors:** F. O. Earle

**Affiliations:** Chicago


ARTICLE XXIX.

### TWO CASES OF ANGULAR CURVATURE, SUPERVENING UPON LATERAL CURVATURE OF THE SPINE.

By F. O. EARLE, M.D., of Chicago.

I herewith send the history of two cases, which present some degree of similarity in the phenomena attending them, and are interesting not only on that account, but also as illustrating one or two points in the differential diagnosis of "Potts' disease" of the spine, exhibiting the slow and insidious access of this malady in certain instances, and showing the necessity of a careful and thorough examination, and the great importance of an early and correct diagnosis in these cases.

I have no recollection of seeing the report of similar cases, and they are the first that have come under my own observation.

CASE I. In April, 1865, I was called to see Miss W., *æt.* 19. She said she had always been delicate; she had a very poor appetite; menstruation irregular, always painful, sometimes profuse, on other occasions almost wanting; bowels generally very loose, though sometimes constipated; she also complained of a burning, painful sensation at various points along the course of the spine, which was greatly increased by the slightest amount of pressure, (*spinal irritation*, so-called,) and examination revealed the existence of a well-marked lateral curvature. She had, moreover, a very pale countenance, constantly complained of exhaustion, her temper was rather irritable, and every movement was indicative of extreme languor.

Regarding all these symptoms as the result of the manifest debility of the organic system, consequent upon an increased irritability of the nervous system, the case was treated in the following manner:—Attendance at school was prohibited, and rest from bodily exertion and mental effort insisted upon. A general tonic course of treatment, in connection with a system of "localized exercise," given daily, was adopted. At the end of two months, my patient had improved in every respect. The painful sensitiveness of the back was entirely relieved; the curvature had almost entirely disappeared; and all the organic functions were performed without that distress and uneasiness by which their actions were formerly characterized.

At this time, she went into the country, and after an absence of a few weeks, returned still more improved, declaring that she was "enjoying perfect health," which appeared to be the case.

In the latter part of April, 1866, I was requested, by the young lady's mother, to see her again. She said the curvature had increased, and, on examination, I discovered that such was *apparently* the case, though a more critical survey disclosed a very slight projection backward of one spinous process in the centre of the supposed curve. This appearance led me to inquire if the patient had suffered any from *pain in the region of the stomach*, and I learned that she was then taking remedies, prescribed by the medical attendant of the family, for dyspepsia, which it was supposed was causing the painful sensations experienced by the patient in the epigastric region. I then requested the patient to stretch the arms above the head and bend forward without flexing the spine, while, at the same time, I grasped the hips firmly. By this means, the *lateral deviation* disappeared, though the projection still remained, leaving no room to doubt the true nature of the case.

I immediately explained the nature and result of the malady to my patient, and, at her mother's request, applied a proper mechanical support. Her vitality being very low at the time, she showed scarcely any signs of improvement until about two weeks since. There is now an amelioration of all the constitutional and local symptoms.

It may be questioned by some reader of this article, whether ulcerative inflammation of the bodies of the vertebræ or of the inter-vertebral substance was not present when this patient was first seen. I answer, no! if such had been the case, all the symptoms would have been aggravated, rather than mitigated, by the treatment at that time adopted.

Case II. Miss B. *æt.* 23, very tall and slim, presented herself, two years ago, for advice with regard to lateral curvature. She complained of nothing in particular, excepting "want of strength." Examination showed lateral curvature in its first stage—there being a lateral bending of the spine low down. A simple apparatus, made of firm webbing and applicable to such cases, was applied, and certain specific and general exercises were given to develope the dorsal muscles, and with such good effect that she was dismissed in a few weeks, greatly benefited.

A short time ago, she returned, at the request of the family physician, saying that the curve was increasing. The statement was doubted, it being so unusual for a lateral curvature to be developed at that age. But a physical examination disclosed the fact that it *was* increasing. Upon minute inquiry being made into the accompanying symptoms, it was found that there *was difficulty of turning or rising after lying down, particularly in the morning;* and a *dull, scarcely noticeable pain in the stomach, especially when she was fatigued.* By stretching the arms above the head and bending forward, as in the other case, the 1st, 2d, and 3d lumbar vertebræ were seen protruding in the manner that is usual when there is loss of substance in the bodies of the vertebræ. In connection with this projection, was a recession of the last dorsal vertebræ.

An apparatus was applied, as in the first case, and with even happier results, the lateral deviation disappearing at once.

